# Human nuclear receptors (NRs) genes have prognostic significance in hepatocellular carcinoma patients

**DOI:** 10.1186/s12957-021-02246-x

**Published:** 2021-04-30

**Authors:** Guangtao Sun, Kejian Sun, Chao Shen

**Affiliations:** Department of Hepatobiliary Surgery, ZiBo Central Hospital, No. 54, Gongqingtuanxi Road, Zibo, Shandong 255036 People’s Republic of China

**Keywords:** HCC, Human nuclear receptors (NRs), Risk score, Prognosis

## Abstract

**Background:**

Hepatocellular carcinoma (HCC) is the third leading cause of cancer-related mortality in the world.

**Method:**

We downloaded the mRNA profiles and clinical information of 371 HCC patients from The Cancer Genome Atlas (TCGA) database. The consensus clustering analysis with the mRNA levels of 48 nuclear receptors (NRs) was performed by the “ConsensusClusterPlus.” The univariate Cox regression analysis was performed to predict the prognostic significance of NRs on HCC. The risk score was calculated by the prognostic model constructed based on eight optimal NRs. Then multivariate Cox regression analysis was performed to determine whether the risk score is an independent prognostic signature. Finally, the nomogram based on multiple independent prognostic factors was used to predict the long-term survival of HCC patients.

**Results:**

The prognostic model constructed based on the eight optimal NRs (NR1H3, ESR1, NR1I2, NR2C1, NR6A1, PPARD, PPARG, and VDR) could effectively predict the prognosis of HCC patients as an independent prognostic signature. Moreover, the nomogram was constructed based on multiple independent prognostic factors including risk score and tumor node metastasis (TNM) stage and could better predict the long-term survival for 3- and 5-year of HCC patients.

**Conclusion:**

Our results provided novel evidences that NRs could act as the potential prognostic signatures for HCC patients.

**Supplementary Information:**

The online version contains supplementary material available at 10.1186/s12957-021-02246-x.

## Highlights

1. Human nuclear receptors were closely related to the development of HCC patients.
2. Risk score calculated based on the optimal NRs could predict the prognosis of HCC patients as an independent prognostic signature.
3. Nomogram based on multiple independent prognostic factors could better predict the long-term survival of HCC patients.

## Introduction

Hepatocellular carcinoma (HCC), the predominant form of primary liver cancer, is fourth leading cause of cancer-related deaths, which also ranks the fifth when considering all the diseases worldwide [1]. Of which, there are only approximately 20-30% of HCC patients are diagnosed at the early stage, and the majority are diagnosed with unresectable disease at the late stage, even given a poor overall prognosis [2]. Currently, several strategies have been explored to prevent the development of HCC including minimizing the expression of risk factors for chronic liver disease through appropriate vaccination programs, antiviral therapies, and treatment of contributing disease with statins, antidiabetic medications, and aspirin [3, 4]. Despite of a lot of advantages in early diagnosis and multidisciplinary treatment for HCC, the long-term prognosis of HCC remains poor. Therefore, the identification of sensitivity and specific molecular markers in HCC patients is an urgent need for personalized treatment and improvement of clinical efficacy.

Human nuclear receptors (NRs) are ligand-activated transcription factors that participate in several biological processes [5]. In the last years, NRs have been identified as master regulators of broad genetic programs in metazoans [6]. Through binding directly to fat-soluble hormones, vitamins, dietary lipids, heme, and xenobiotic compounds, NRs can regulate multiple genes expression in a variety of cell types [7]. These changes finally lead to ultimately culminate transactivation or trans-repression of target genes, then participate in human multiple diseases [8].

NRs superfamily consists of 48 members that are divided into seven subfamilies including thyroid hormone receptors (class I), retinoid X receptors (class II), estrogen receptors (class III), nerve growth factors (class IV), steroidogenic factors (class V), germ cell nuclear factor (class VI), and class 0 NRs nuclear receptor transcription factors DAX-1 (NR0B1) and short heterodimeric partner (NR0B2) that lack a DNA-binding domain (DBD) [9]. With the progress of scientific research and technology, NRs has been reported to participate in the development and used for the treatment of various cancer. For instance, AR axis-targeting therapeutics such as androgen-deprivation therapy and antiandrogens have been the gold-standard treatments for recurrent or advanced prostate cancer [10]. The lipid-sensors, peroxysome proliferator-activated receptor-γ (NR1C3), liver X receptor-β (NR1H2), and liver X receptor-α (NR1H3) are likely to be onco-suppressors in breast-cancer [11]. The main coactivators nuclear receptor coactivator (NCoA) 1 to 3, NCoA-6, peroxisome proliferator-activated receptor coactivator 1α (PGC1-α), p300, cAMP response element binding protein (CREB) binding protein (CREBBP) and methyl-CpG binding endonuclease (MED1), and corepressors nuclear receptor co-repressor (N-CoR) 1 and 2, nuclear receptor-interacting protein (NRIP1) and metastasis-associated protein 1 (MTA1) of nuclear receptors have been identified to contribute to the treatment of colorectal cancer [12]. Previous studies have reported that NRs are master transcriptional regulators of hepatocellular development, differentiation and function; meanwhile, NRs have been implicated in the modulation of hepatocyte priming and proliferation in regenerating liver, chronic hepatitis, and HCC development [13]. However, the prognostic significance of NRs in HCC patients has not been well studied.

In this study, we analyzed the expression of 48 NRs in HCC samples, and found that NRs were closely related to the development of HCC patients, and also explore the prognostic significance of NRs on the HCC patients. Our study provided new evidences that NRs have potential prognostic significance of HCC patients.

## Materials and methods

### Datasets

On the one hand, we downloaded mRNA expression profiles and clinical information of 371 HCC patients from The Cancer Genome Atlas database (TCGA, https://tcga-data.nci.nih.gov/tcga/), of which, 365 patients have the complete survival information. The all cancer samples of 365 patients were randomly divided into training set (*N* = 245) and testing set (*N*= 123), and the corresponding clinical information including these 365 patients with complete survival information, training set, and testing set was shown in Table 1. On the other hand, in order to verify the prognostic model based on nuclear receptor, we also downloaded mRNA expression profiles and clinical information of 237 HCC patients from International Cancer Genome Consortium database (ICGC, https://icgc.org/) with number of Liver Cancer-RIKEN, JP (LIRI-JP).

**Table 1:** TCGA 365 HCC patients clinicopathological characteristics

Characteristics	Groups	Patients			*X*^2^	*p* value
		Total (*N* = 365)	Training cohort (*N* = 245)	Testing cohort (*N* =122)		
		Number	Number	Number		
Sex	Male	246	159	89	2.4096	0.2997
	Female	119	86	33		
Age at diagnosis	Median	61	61	61	0	1
	Range	16-90	16-85	20-90		
Pathological TNM stage	I	170	117	54	0.8923	0.9988
	II	84	55	30		
	III	83	55	28		
	IV	4	2	2		
	Unknown	24	16	8		
Histologic grade	G1	55	35	20	9.1377	0.3308
	G2	175	112	64		
	G3	118	82	37		
	G4	12	12	0		
	Unknown	5	4	0		
Vital status	Alive	234	154	82	0.6755	0.7134
	Dead	131	91	40		
Adjacent hepatic tissue inflammation extent type	None	117	75	43	2.774	0.8366
	Mild	98	63	36		
	Severe	17	11	6		
	Unknown	133	96	37		
Person neoplasm cancer status	Tumor free	161	110	52	0.1727	0.9965
	With tumor	122	81	42		
	Unknown	82	54	28		
Vascular tumor cell type	None	205	144	62	3.1067	0.7953
	Micro	90	60	31		
	Macro	16	9	7		
	Unknown	54	32	22		
Race	Asian	155	106	50	2.6951	0.952
	American Indian or Alaskan native	1	1	0		
	Black or African American	17	13	4		
	White	182	120	63		
	Unknown	10	5	5		
Sample type	Primary tumor	364	244	121	0.6672	0.7164
	Recurrent tumor	1	1	1		

### Human nuclear receptors

Early phylogenetic studies further classified the NR superfamily into seven subfamilies or classes based on sequence similarity, including thyroid hormone receptors (class I), retinoid X receptors (class II), estrogen receptors (class III), nerve growth factors (class IV), steroidogenic factors (class V), germ cell nuclear factor (class VI), and class 0 NRs (NR0B1 and NR0B2) that lack a DBD [14]. In this study, we performed the analysis based on mRNA of 48 human nuclear receptors, and the information of 48 NRs was shown in Additional file 1: Table S1.

### Analysis of consensus clustering

The consensus clustering analysis was performed by “ConsensusClusterPlus” function package in R language [15] based on mRNA expression levels of 48 NRs in HCC samples. Meanwhile, principle component analysis (PCA) was also performed.

### LASSO Cox regression analysis

Univariate Cox regression analysis was performed on all HCC samples based on the mRNA levels of 48 NRs, with *p* < 0.05 was the significant threshold to screen NRs which were significantly related to prognosis of HCC patients. LASSO Cox regression analysis was then performed to select the optimal NRs using the glmnet package in R language [16]. Next, risk score of each sample was calculated based on optimal NRs through below formula:

$$ \mathrm{Risk}\ \mathrm{score}={\sum}_{\mathrm{i}=1}^{\mathrm{n}}\mathrm{Co}{\mathrm{ef}}_{\mathrm{i}}\ast {\mathrm{x}}_{\mathrm{i}}, $$

Of which, Coefi is risk coefficient of each NR calculated by LASSO Cox regression analysis and Xi is mRNA level of NR in this study. Then the optimal cutoff value of the risk score was determined based on survival (https://cran.r-project.org/web/packages/survival/), survminer (https://cran.r-project.org/web/packages/survminer) package of R language, and bilateral log-rank test. The HCC patients were divided into low risk group and high risk group according to the optimal cutoff value.

### Survival analysis

The overall survival rate of different groups was analyzed using survival package and survminer package in R language based on Kaplan-Meier method, and the significance of the survival rate in different groups was analyzed by log-rank method. The time-dependent receiver operating characteristic (ROC) curves of HCC samples were drawn by use of survivalROC package in R language [17]. Multivariate Cox regression model was used to evaluate whether the risk score calculated can predict the survival of HCC patients independent of other factors.

### Infiltration proportion of immune cells

The relative proportion of 22 immune cells in each HCC cancer sample was calculated by the CIBERSORT software [18]. CIBERSORT is a method for characterizing cell composition of immune cells with 547 preset barcode genes based on the deconvolution algorithm according to their gene expression profiles, and the sum of ratios of all estimated immune cell types in each sample is 1.

### The construction of nomogram

Nomograms are widely used for cancer prognosis because of their ability to reduce statistical predictive models into a single numerical estimate of the probability of an event, such as death or recurrence, that is tailored to the profile of an individual patient [19]. To predict the survival probability of HCC patients at 1 year, 3 years, and 5 years, we constructed a nomogram by RMS package (https://cran.r-project.org/web/packages/rms) of R language based on all independent prognostic factors determined by Multivariate Cox regression analysis, and plotted the calibration curve of nomogram to determine the relationship between the predicted probability of nomogram and the actual incidence.

### Statistical analysis

The overall survival rate of samples was estimated by Kaplan-Meier method, and the significance of the difference in survival rate among different groups was analyzed by log-rank method. Wilcoxon rank-sum tests were used to compare the differences of infiltration of immune cells between different sample groups, with *p* < 0.05 as the significant threshold. Statistical analysis was performed with R software v3.5.2.

## Results

### The human nuclear receptors (NR) could effectively separate HCC samples with different prognosis

**Fig. 1:**
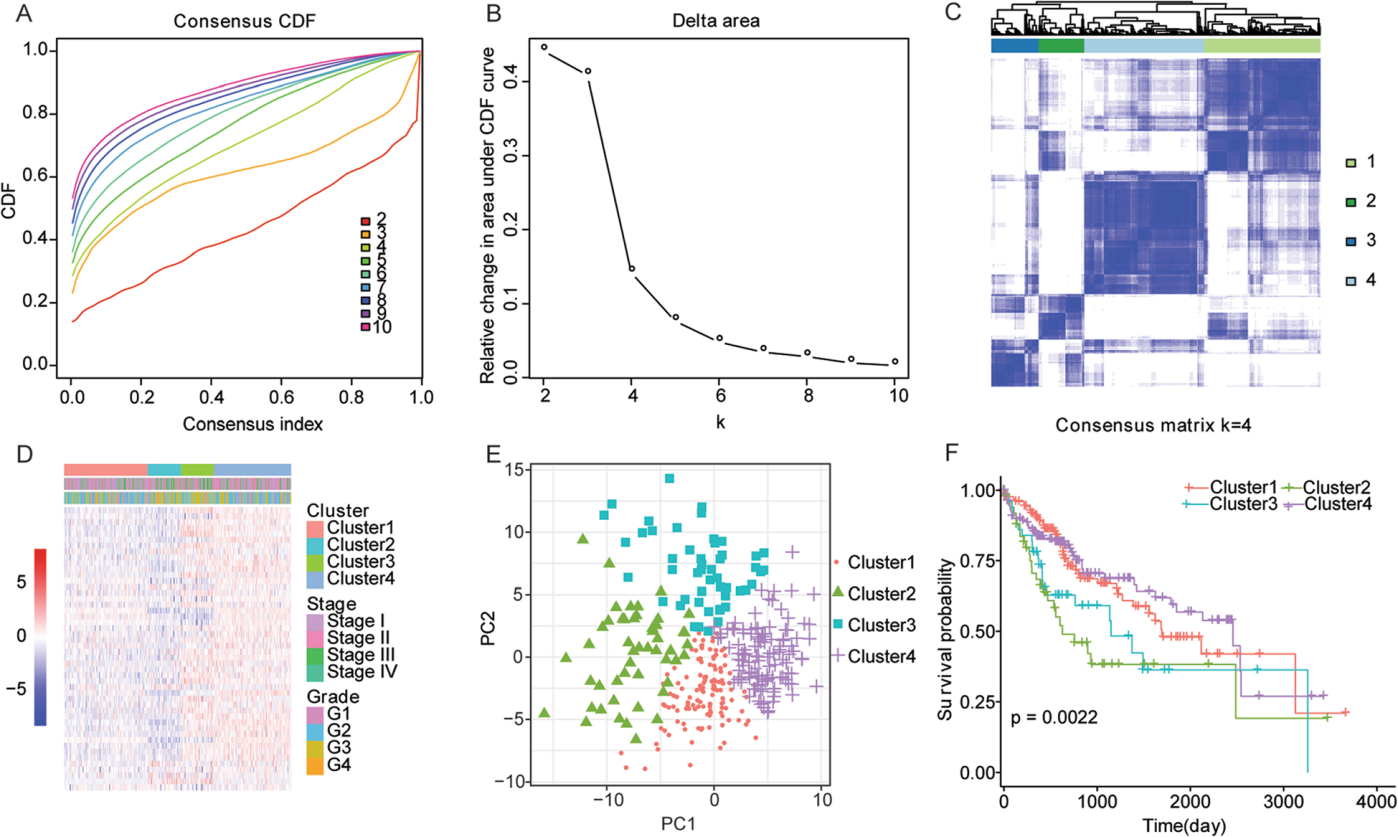
The consensus clustering analysis of HCC samples based on mRNA levels of human nuclear receptors. **a** The cumulative distribution function (CDF) of clustering (*k*, 2-10). **b** The relative changes of the area in the CDF curve (*k*, 2-10). The horizontal axis is the numbers of clusters (*k*), and the vertical axis is the relative changes of the area in the CDF curve. **c** The consistency matrix of sample with *k* = 4. **d** The heatmap of the expression of NRs in the four types of samples. The rows represent genes, the columns represent samples, red indicates high expression, blue indicates low expression, and the categories of samples are marked with different colors on the top of the heatmap. **e** The principle component analysis (PCA) of HCC samples. The points of different colors represent samples of different groups. The distance among the points is closer, the expression of NRs is more similar. **f** The Kaplan-Meier survival curve of HCC patients. The horizontal axis represents time, the vertical axis represents survival rate, and the color represents different groups. *P* value is calculated by log-rank test

Based on the cumulative distribution function of clustering (Fig. 1a and b), we performed consensus clustering analysis with the mRNA levels of 48 NRs by using the “ConsensusClusterPlus” function package in R language, and divided all HCC samples into four categories (*k* = 4). The consistency matrix (Fig. 1c) and the heatmap of consensus matrix (Fig. 1d) all showed that the consistency clustering based on the mRNA level of NRs could clearly distinguish these four categories. The results of PCA analysis also showed that the differences among the four groups of samples were significant (Fig. 1e). The survival analysis based on Kaplan-Meier method was performed and indicated that there were significant differences in overall survival among the four types of samples, and cluster3 exhibited a worst prognosis (Fig. 1f). These results indicated that the mRNA level of NRs could efficiently separate HCC samples with different prognosis.

### Prognostic significance of NRs in HCC

**Fig. 2:**
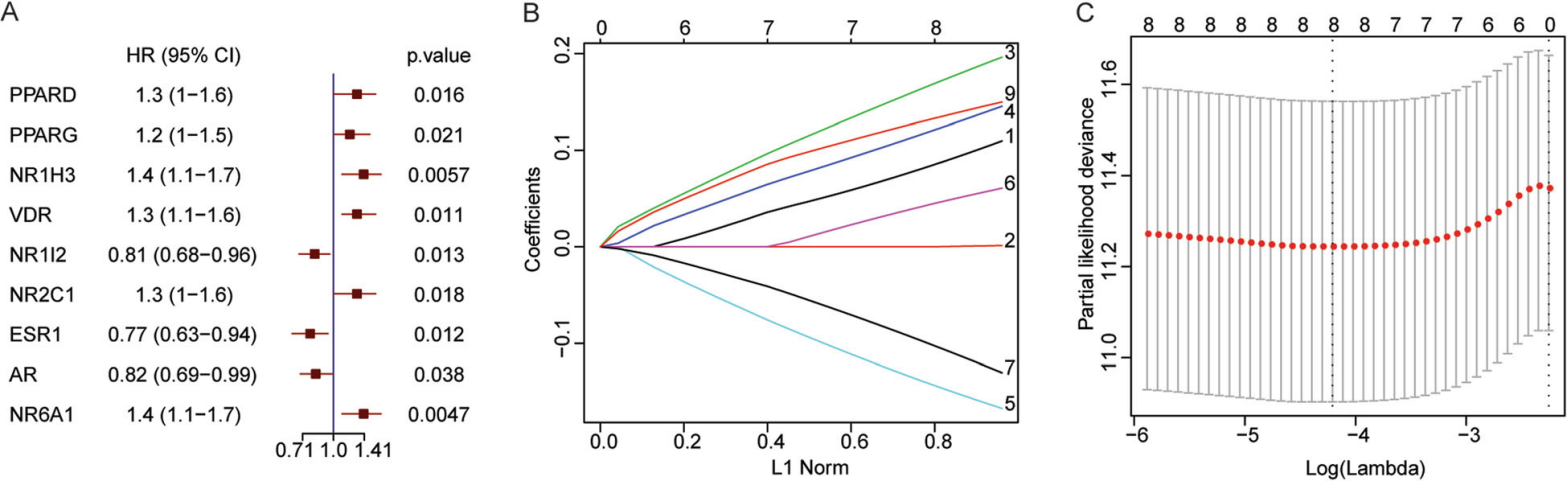
The construction of the prognostic model for HCC. **a** The forest plot of univariate Cox regression analysis with 9 NRs which were significantly related to prognostic value of HCC patients. HR represents hazard ratio and 95% CI is the 95% confidence interval. **b** The coefficient spectrum of LASSO Cox regression model. **c** The tuning parameter lambda was determined by LASSO regression analysis. The horizontal axis is log(lambda), and the vertical axis is partial likelihood deviance. The optimal lambda value after taking the log below the dotted line, and the number of variables is corresponding to the top of the optimal lambda

In order to determine the prognostic role of NRs in HCC, the univariate Cox regression analysis with training set samples based on the mRNA level of 48 NRs was performed, and the hazard ratio (HR) of each NR was calculated with *p* < 0.05 as the significant threshold. The results indicated that these six NRs including peroxisome proliferator-activated receptor delta (PPARD) (HR = 1.3, 95% CI: 1-1.6, *p* = 0.016), peroxisome proliferator-activated receptor gamma (PPARG) (HR = 1.2, 95% CI: 1-1.5, *p* = 0.021), NR1H3 (HR = 1.4, 95% CI: 1.1-1.7, *p* = 0.0057), vitamin D receptor (VDR) (HR = 1.3, 95% CI: 1.1-1.6, *p* = 0.011), testicular receptor 2 (NR2C1) (HR = 1.3, 95% CI: 1-1.6, *p* = 0.018), and germ cell nuclear factor (NR6A1) (HR = 1.4, 95% CI: 1.1-1.7, *p* = 0.0047) were significantly related to the overall survival in HCC samples, and were risk genes that can result in the poor prognosis (Fig. 2a). Meanwhile, the three NRs including pregnane X receptor (NR1I2) (HR = 0.81, 95% CI: 0.68-0.96, *p* = 0.013), estrogen receptor α (ESR1) (HR = 0.77, 95% CI: 0.63-0.94, *p* = 0.012), and androgen receptor (AR) (HR = 0.82, 95% CI: 0.69-0.99, *p* = 0.038) were also significantly related to the overall survival in HCC samples, but these three NRs were protective genes that can be favorable for prognosis (Fig. 2a). Then, LASSO Cox regression analysis on training set samples based on the selected 9 NRs was performed, eight optimal NRs (NR1H3, ESR1, NR1I2, NR2C1, NR6A1, PPARD, PPARG, and VDR) were determined based on the lowest lambda value of each gene (Fig. 2b and c).

**Fig. 3:**
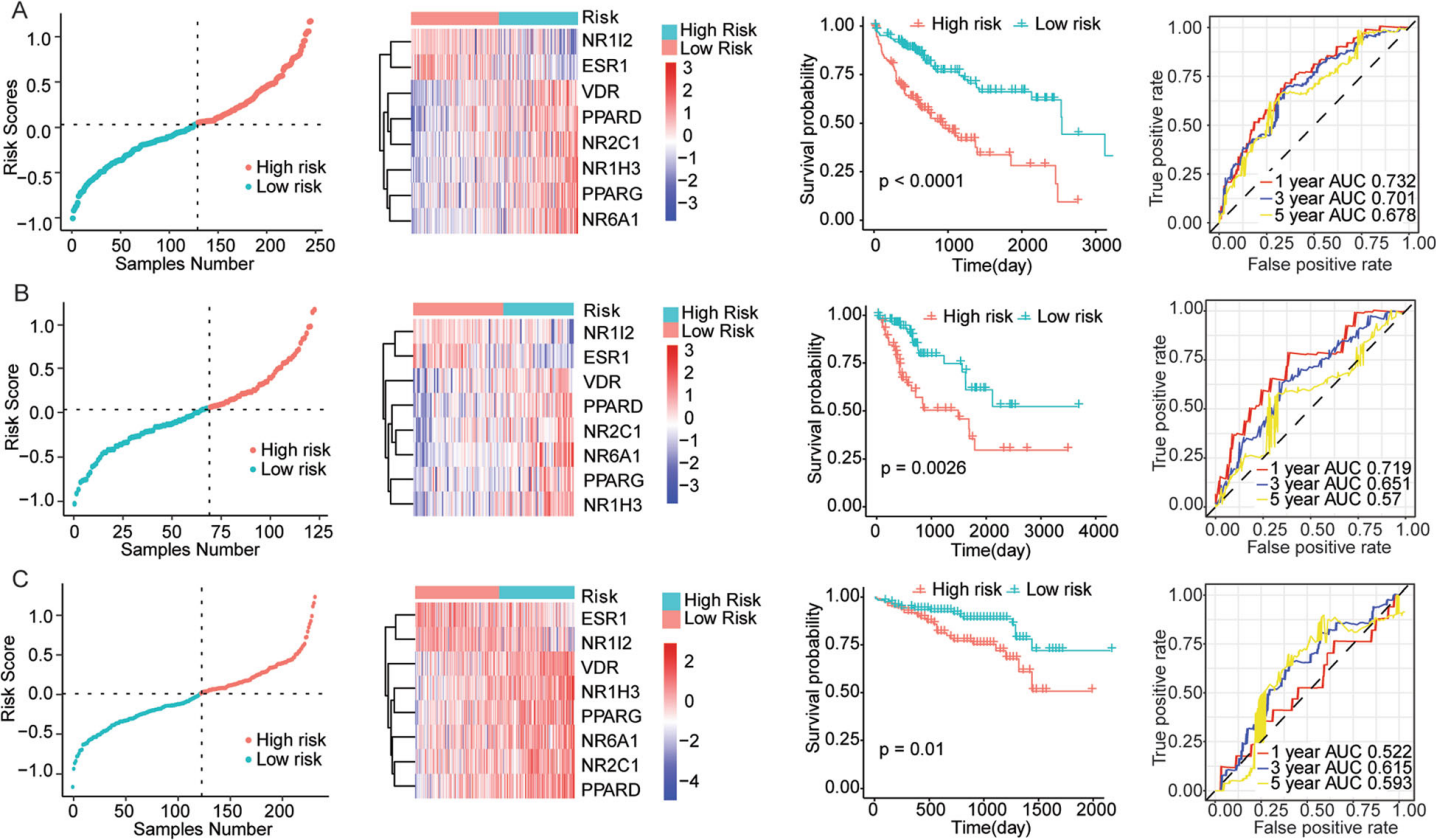
The prognostic model could efficiently predict the survival of HCC patients. **a** The distribution of risk scores of TCGA training set samples on the left. A point represents a sample, the red point indicates the sample with high risk score, the green point indicates the sample with low risk score, and the intersection point is the optimal risk score; the clustering heatmap of mRNA expression of selected NRs in the TCGA training set on the second left. The rows represent genes, the columns represent samples, red indicates high expression, blue indicates low expression, and the categories of samples are marked with different colors on the top of the heatmap; the Kaplan-Meier survival curve of the TCGA training set was performed on the third left. The horizontal axis represents time, the vertical axis represents survival rate, and the color represents different groups; the time-dependent ROC curve of TCGA training set on the right. The horizontal axis represents false positive, and the vertical axis represents true positive. The accuracy of prediction is evaluated by the value of AUC (the area under ROC curve). **b** The distribution of risk scores on TCGA testing set samples; the clustering heatmap of mRNAs expression on TCGA testing set samples; the Kaplan-Meier survival curve and the time-dependent ROC curve on TCGA testing set samples. **c** The distribution of risk scores on TCGA testing set samples; the clustering heatmap of mRNAs expression on TCGA testing set samples; the Kaplan-Meier survival curve and the time-dependent ROC curve on ICGC testing set samples

Next, to obtain a uniform threshold to successfully divide all HCC patients into high risk group and low risk group across different sample sets, we standardized the expression values of 8 genes both in the TCGA dataset and ICGC dataset into the values with an average value of 0 and a standard deviation (SD) of 1. Then, we weighted the normalized expression of each nuclear receptor with the regression coefficient of LASSO Cox regression analysis and established a risk score model for predicting patient survival by the following formula: Risk score = 0.1765 × express value of NR1H3 − 0.11 × express value of ESR1 − 0.1501 × express value of NR1I2 + 0.0495 × express value of NR2C1 + 0.1377 × express value of NR6A1 + 0.0917 × express value of PPARD + 0.0004 × express value of PPARG + 0.1276 × express value of VDR. Based on the formula, risk score of each patient was calculated. And the samples of TCGA training set, TCGA testing set, and ICGC verifying set were divided into high-risk group and low-risk group according to the calculated optimal cut-off point (0.0326), the risk score distribution of samples in three data sets was shown in the left panel of Fig. 3. Meanwhile, the expression of eight NR in the model exhibited significant differences between high-risk group and low-risk group (the second from left of Fig. 3). The survival curve showed that the HCC samples of high-risk group had poor overall survival than low-risk group in the three data sets (the third from left of Fig. 3). In addition, the time dependent ROC curve showed that the area under curve (AUC) of 1-year, 3-year, and 5-year survival of the training set is 0.732, 0.701, and 0.678; the AUC of 1-year, 3-year, and 5-year survival of the testing set was 0.719, 0.651, and 0.57; and the AUC of the 1-year, 3-year, and 5-year survival of ICGC verifying set is 0.522, 0.615, and 0.593, respectively (the right panel of Fig. 3). The results indicated that prognostic model constructed based on the eight NRs (NR1H3, ESR1, NR1I2, NR2C1, NR6A1, PPARD, PPARG, and VDR) could effectively predict the prognosis of HCC patients in three sets of data.

### Immune status of HCC samples between the high- and low-risk groups

**Fig. 4:**
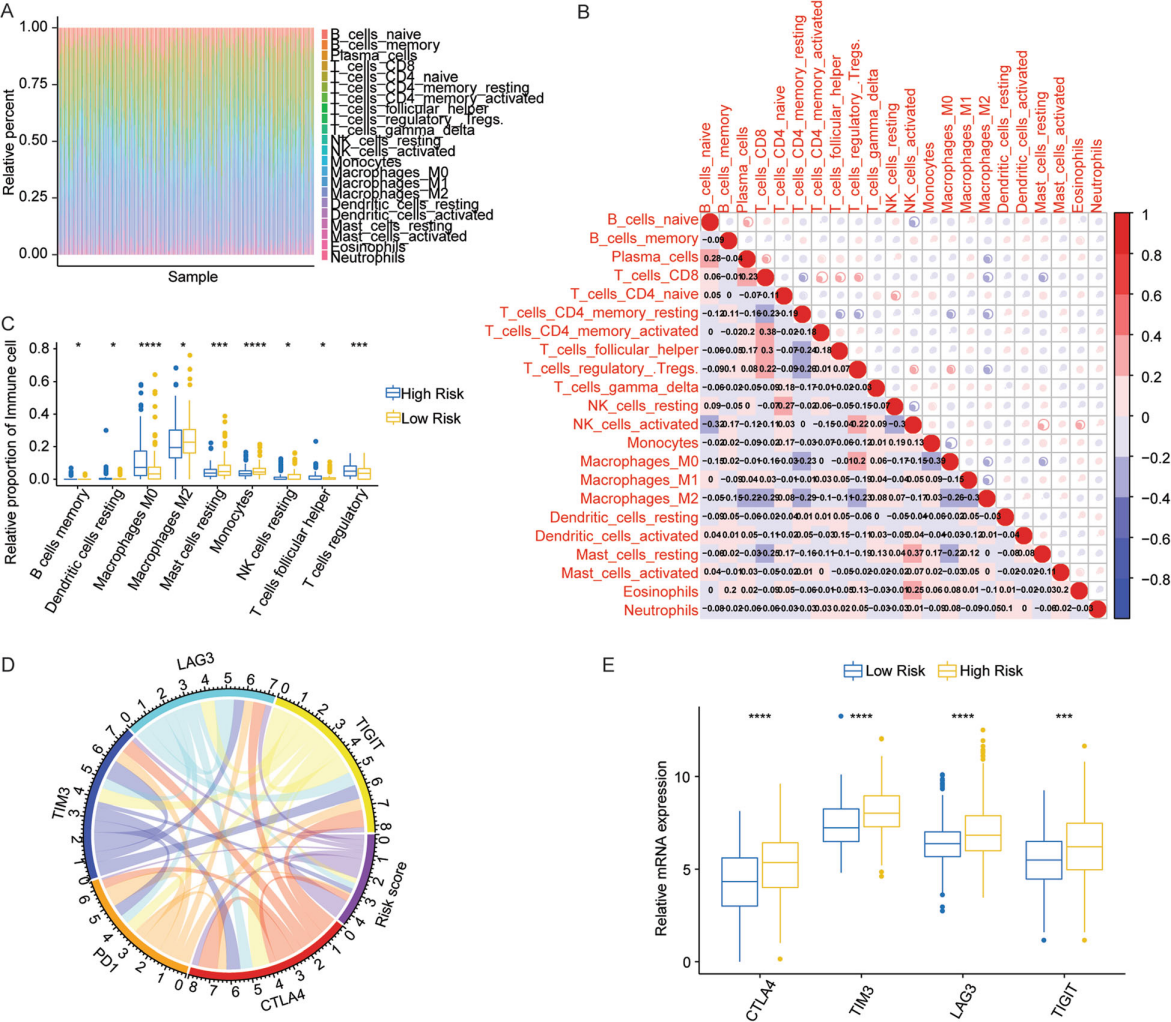
Immune infiltration of HCC samples between the high and low risk groups. **a** The relative proportion of immune infiltration in all HCC patients. **b** Correlation matrix of proportion of 22 immune cells. Red indicates positive correlation, blue indicates negative correlation. The darker the color, the greater the correlation. **c** The boxplot of immune cells with significantly different proportions of infiltration in the high low risk group. The horizontal axis is the type of immune cells, and the vertical axis is the relative infiltration proportion of immune cells. Different colors represent the high or low risk group. **d** The correlative circos between risk score and the expression of five prominent immune checkpoints. **e** The differentially expressed immune checkpoints between the high group and low risk group. *p<0.05, **p<0.01, ***p< 0.001, and ****p< 0.0001

We estimated the differences of the immune infiltration including 22 immune cells in HCC samples between the high- and low-risk groups through comprehensive analysis based on CIBERSORT and LM22 eigenmatrix. The result of immune cells infiltration in 365 patients with HCC was summarized in Fig. 4a, and changes in the proportion of tumor infiltrating immune cells among different patients might represent the intrinsic characteristics of individual differences. Meanwhile, the results of analysis indicated that the proportion of infiltration of different types of immune cells was weakly correlated (Fig. 4b). In addition, there were significant differences in the infiltration proportion of nine types of immune cells including B cells memory, dendritic cells resting, macrophages M0, macrophages M2, mast cells resting, monocytes, NK cells resting, T cells follicular helper, and T cells regulatory (Fig. 4c).

The expression of immune checkpoint has becoming a promising biomarker for the selection of immunotherapy for liver cancer patients [20]. We found a close correlation between risk score of HCC patients and the expression of key immune checkpoints composed of cytotoxic T lymphocyte-associated protein 4 (CTLA4), programed death-1 (PD1), T cell immunoglobulin mucin 3 (TIM3), lymphocyte activation gene 3 (LAG3) and T cell immunoreceptor with Ig, and ITIM domains (TIGIT) (Fig. 4d). Meanwhile, expression of CTLA4, PD1, TIM3, LAG3, and TIGIT in HCC patients between the high- and low-risk groups were analyzed, and the results indicated that the expression of CTLA4, TIM3, LAG3, and TIGIT in high-risk group were obviously lower than low-risk group (*p* < 0.05) (Fig. 4e), suggesting that the poor prognosis of HCC patients with high risk might be due to the immunosuppressive microenvironments in liver cells.

### Risk score is an independent prognostic signature for HCC patients

**Fig. 5:**
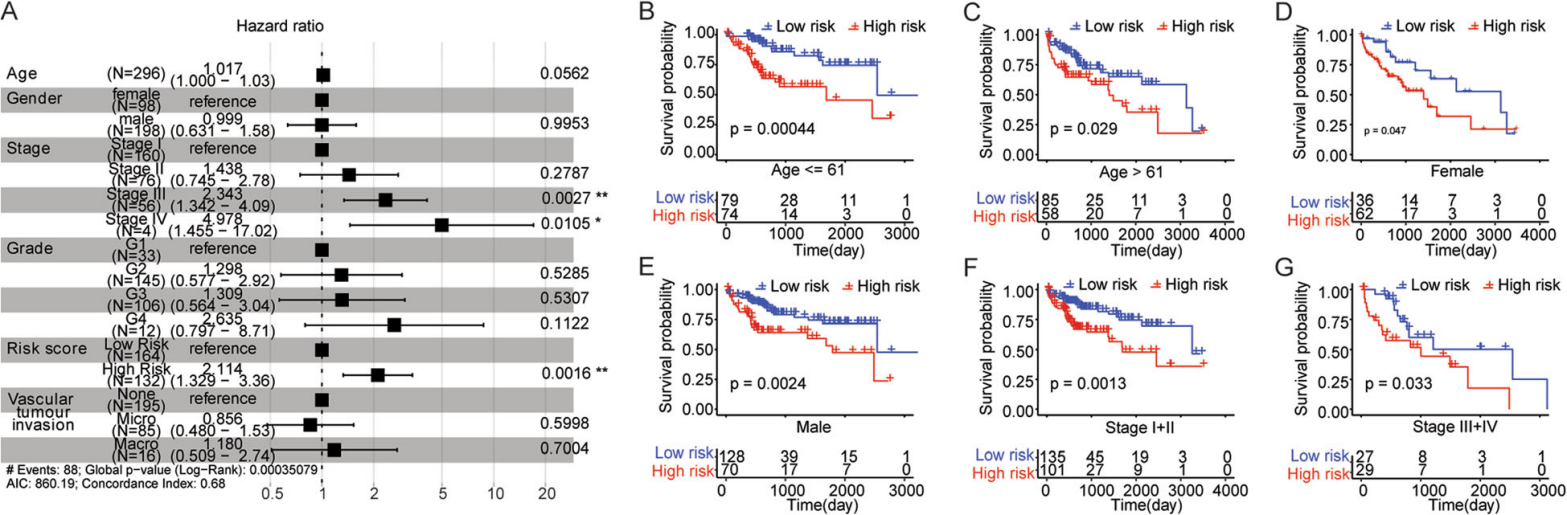
Risk score is an independent prognostic signature for HCC. **a** The forest plot of multivariate Cox regression analysis. Compared with reference samples, samples (hazard ratio > 1) have a higher risk of death, while samples (hazard ratio < 1) have a lower risk of death. **b**-**f** The Kaplan-Meier survival curves of HCC samples with different clinicopathologic factors. The horizontal axis represents time, the vertical axis represents survival rate, and the color represents different groups. *P* value is calculated by log-rank test

The multivariate Cox regression analysis was performed to determine whether the risk score is an independent prognostic signature bringing into multiple factors including age, gender, tumor node metastasis (TNM) stages, grade, vascular tumor invasion and risk score. The results indicated that risk score was still significantly correlated with overall survival (Fig. 5a). Meanwhile, the samples with high risk score had a greater risk of death, which was an adverse prognostic factor with the low-risk group as a reference (HR=2.114, 95% CI: 1.329-3.36, *p* =0.0016) (Fig. 5a).

To further explore the prognostic value of risk score in HCC samples with different clinicopathologic factors including age, gender, and TNM stage, we regrouped HCC samples according to these above factors and performed Kaplan-Meier survival analysis. For samples of age ≤ 61 (Fig. 5b), samples of age > 61 (Fig. 5c), female samples (Fig. 5d), male samples (Fig. 5e), samples of early cancer (stage I+II) (Fig. 5f), and samples of late cancer (stage III+IV) (Fig. 5g), the overall survival in the high-risk group was significantly lower than that in the low-risk group, indicating that risk score could predict the prognosis of patients with HCC as an independent prognostic signature.

### The nomogram model could efficiently predict the long-term survival of HCC patients

**Fig. 6:**
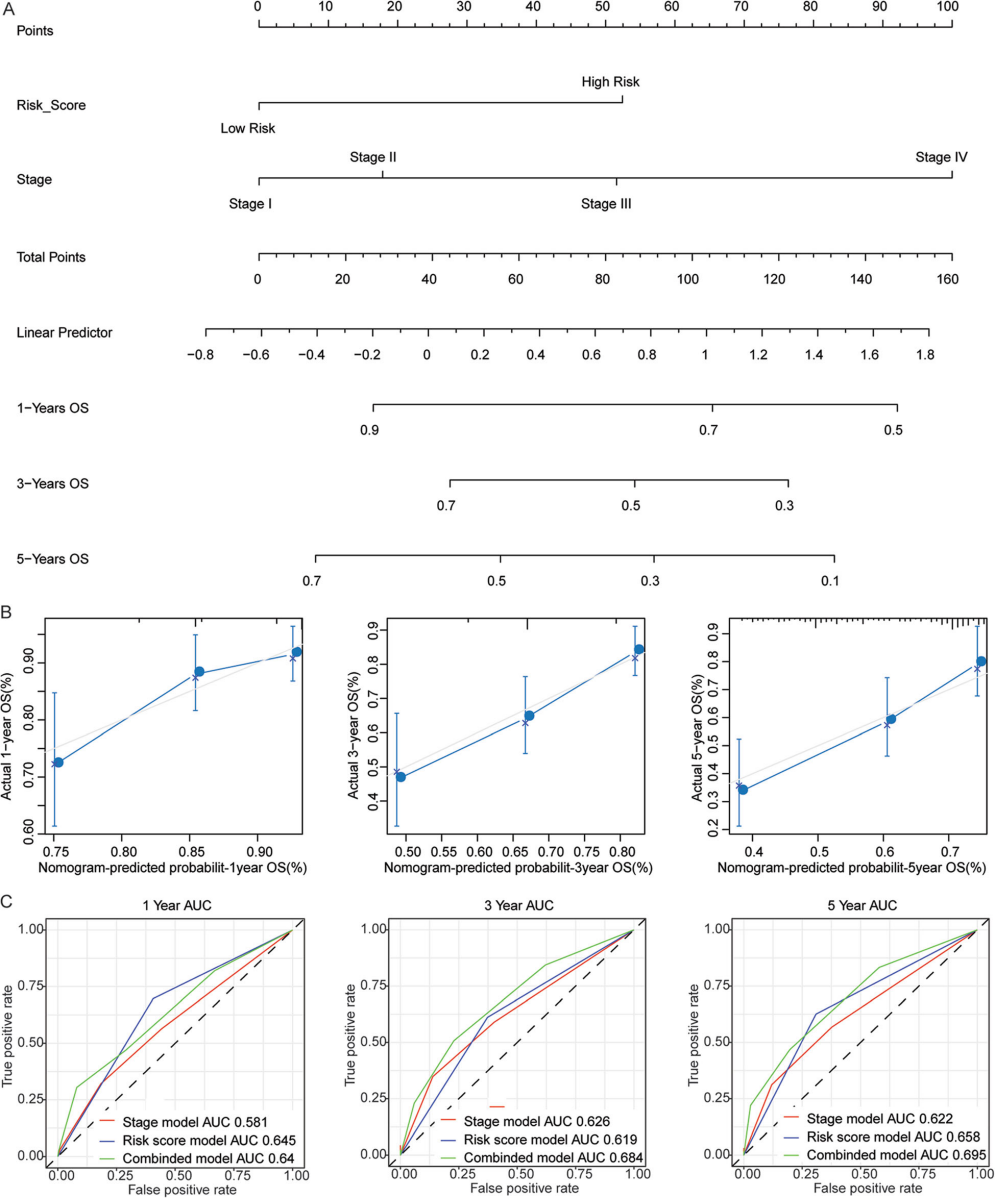
The nomogram could efficiently predict the survival of HCC patient. **a** The nomogram for predicting the probability of 1-, 3-, and 5-year overall survival in HCC patients. **b** The calibration curves of overall survival probabilities of 1, 3, and 5 year was predicted by nomogram in HCC patients. The horizontal axis represents survival rate predicted by nomogram, the vertical axis represents actual survival rate. **c** The time-dependent ROC curves of 1-, 3-, and 5-year overall survival probabilities for HCC patients was predicted by nomogram

We constructed the nomogram model based on the two independent prognostic factors, TNM stage and risk score (Fig. 6a). For each patient, we draw three lines up to determine the point which was obtained from each factor in the nomogram. The sum of these points was located on the “Total Points” axis, and then a line is drawn down from the “Total Points” axis to determine the probability of the survival for 1, 3, and 5 years in HCC patients. The results indicated that the corrected curve is close to the ideal curve (a 45-degree line with slope of 1 through the origin of the coordinate axis), suggesting that the prediction is in better agreement with the actual results (Fig. 6b). And compared with nomogram containing one independent prognostic factor, the nomogram containing all independent prognostic factors had the largest AUC of survival for 3 year or 5 year in HCC patients (Fig. 6c), indicating that the nomogram constructed based on the all independent prognostic factors could efficiently predict the long-term survival of HCC patients compared with the nomogram based on a single independent prognostic factor.

## Discussions

Liver is considered as an extremely important organ, and even small abnormity could lead to severe dysfunction in the body. For example, the homeostasis of bile acid is indicated to be related to cholestatic liver disease [1]. HCC is the most common type of primary liver cancer, with more than 700,000 deaths each year worldwide [21]. The occurrence of HCC can be attributed to various distinct risk factors such as excessive chronic alcohol intake and dietary exposure to aflatoxin, and chronic liver diseases including chronic infection with hepatitis B (HBV) or C (HCV) virus, often result in liver cirrhosis, which is a critical risk factor for the progression of HCC [22–25]. And hepatitis infection is associated with not only liver disease but also thalassemia [26, 27]. Chronic liver injury is associated with the abnormal regulation of hepatocytes growth, leading to dysplastic and regenerative nodules and tumorigenesis of HCC [25]. In the past decades, though numbers of treatments have been identified for HCC patients including surgery, chemotherapy, and radiotherapy, the mortality rate is still higher [28, 29]. Meanwhile, the high metastasis and recurrence rates of HCC illustrate that the overall prognosis of HCC remains unsatisfactory [30]. Substantial efforts have been devoted to investigate HCC-related biomarkers. Bai et al. established a predictive prognostic model of HCC with the apoptosis-related genes [31]. Li et al. identified eight pivotal genes which were associated with the HCC occurrence [32]. However, there is still an urgent need to identify efficient prognosis-related factors or prognostic predictors to further improve the clinical treatment of HCC.

Human nuclear receptors (NRs) have been reported to act as new therapeutic targets for various cancers also including HCC. The estrogen receptor (ER) is predominantly expressed between malignant and normal liver cells, while the expression of ER difference between males and females, and ER could be targeted for designing HCC therapy [33]. Transforming growth factor β (TGF-β) consists of several proteins which have related structures, such as TGF-β, bone morphogenic proteins, and activins/inhibins, and is closely associated with various cellular functions, for example, proliferation, migration, and apoptosis [34]. Moreover, TGF-β also plays an important role in cancer, which exerts inhibiting effects in the early stage by suppressing the progression of cell cycle and enhancing apoptosis, while accelerating the development of tumor in the late stage by promoting the invasiveness and metastasis of tumor accompanied by the effects of epithelial to mesenchymal transition (EMT) [35]. TGF-β induced chemoresistance in liver cancer is modulated by xenobiotic nuclear receptor PXR [36]. Although there was no association between VDR polymorphisms with HBV infection risk, the ApaI polymorphism might be a genetic factor associated with the clinical outcome and disease progression in HBV infected patients [37]. Khan et al. has also reported that NLRP12 plays a critical role in suppressing the progression of HCC via negative regulation of the JNK pathway [38]. These reports all indicated that human nuclear receptors were closely associated with development of HCC and could be novel therapeutic targets. Here, we performed consensus clustering analysis with the mRNA levels of 48 NRs and divided all HCC samples into four categories, and found that the consistency clustering based on NRs could clearly distinguish these four categories.

Next, univariate Cox regression analysis and LASSO Cox regression analysis was performed to select the optimal eight NRs which were significantly related to the progression of HCC. Risk score calculated by prognostic model constructed based on the eight optimal NRs (NR1H3, ESR1, NR1I2, NR2C1, NR6A1, PPARD, PPARG, and VDR) could effectively predict the prognosis of HCC patients. Immune checkpoints include stimulatory and inhibitory checkpoint molecules, and Xu et al. has summarized current knowledge and recent developments in immune checkpoint-based therapies for the treatment of hepatocellular carcinoma [20]. We also analyzed the expression of immune checkpoints and found that there was a close correlation between risk score of HCC patients and the expression of key immune checkpoints including CTLA4, PD1, TIM3, LAG3, and TIGIT. These suggested that the poor prognosis of HCC patients with high risk might be due to the immunosuppressive microenvironments in liver cells.

Meanwhile the multivariate Cox regression analysis has demonstrated that risk score could predict prognostic significance for HCC patients as an independent prognostic signature. Finally, the nomogram based on the two independent prognostic factors, TNM stage and risk score, could better predict the overall survival of HCC compared with that based on a single independent prognostic factor.

There were some limits existing in this study: (1) more HCC samples were used to verify our prognostic model and nomogram model. (2) Further specific experiments were needed to determine the close relationship between NRs and the development of HCC, as well as the prognostic significance of HCC.

## Conclusions

In summary, we found that the expression of human nuclear receptors (NRs) was closely related to the development of HCC patients. Risk score calculated by the prognostic model constructed in this study could efficiently predict the prognosis of HCC patients as an independent prognostic signature. Meanwhile, the nomogram based on multiple independent prognostic factors including risk score and TNM stage could better predict the long-term survival for 1, 3, and 5 years of HCC patients.

## Supplementary Information

**Additional file 1: Table S1.** The list of 48 NRs used in this study.

## Data Availability

All data generated or analyzed during this study are included in this published article.
