# Supplementary material for: Human nuclear receptors (NRs) genes have prognostic significance in hepatocellular carcinoma patients
Source: World J Surg Oncol. 2021 Apr 30;19:137. doi: 10.1186/s12957-021-02246-x (PMC8091722; doi:10.1186/s12957-021-02246-x)
Supplement: Supplementary file 1 — Additional file 1: Table S1. The list of 48 NRs used in this study. [file 12957_2021_2246_MOESM1_ESM.docx]

Table S1 The list of 48 NRs used in this study.

| Gene symbol and full name | | Gene abbreviation | NRNC Symbol | NR categorya | Receptor | Ligand(s)b | Dimerizationc | Associated cancer form(s)a |
| --- | --- | --- | --- | --- | --- | --- | --- | --- |
| *Class I: thyroid hormone receptor-like* | | | | | | | | |
| *THRA* | Thyroid hormone receptor-α | *THRα* | NR1A1 | Endocrine | Thyroid hormone receptor | Thyroxine (T4), Triiodothyronine (T3) | Heterodimer/ monomer | KIRC/KIRP |
| *THRB* | Thyroid hormone receptor-β | *THRβ* | NR1A2 | Endocrine |  |  |  |  |
| *RARA* | Retinoic acid receptor-α | *RARα* | NR1B1 | Endocrine | Retinoic acid receptor | All-trans and 9-cis retinoic acid | Heterodimer | BRCA, COAD, SKCM |
| *RARB* | Retinoic acid receptor-β | *RARβ* | NR1B2 | Endocrine | COAD, SKCM |  |  |  |
| *RARG* | Retinoic acid receptor-γ | *RARγ* | NR1B3 | Endocrine | BRCA, COAD, SKCM |  |  |  |
| *PPARA* | Peroxisome proliferator activated receptor-α | *PPARα* | NR1C1 | Adopted | Peroxisome proliferator activated receptor | Fatty acids | Heterodimer | KIRC/KIRP |
| *PPARD* | Peroxisome proliferator activated receptor-β/δ | *PPARδ* | NR1C2 | Adopted | KIRC/KIRP |  |  |  |
| *PPARG* | Peroxisome proliferator activated receptor-γ | *PPARγ* | NR1C3 | Adopted | HNSC, LIHC, COAD, LUAD/ LUSC, KIRC/KIRP, SKCM |  |  |  |
| *NR1D1* | Rev-ErbAα | *REVERBα* | NR1D1 | Adopted | Rev-ErbA | Heme | Monomer/ homodimer |  |
| *NR1D2* | Rev-ErbAα | *REVERBβ* | NR1D2 | Adopted |  |  |  |  |
| *RORA* | RAR-related orphan receptor-α | *RORα* | NR1F1 | Adopted | RAR-related orphan receptor | Oxysterols | Monomer | HNSC |
| *RORB* | RAR-related orphan receptor-β | *RORβ* | NR1F2 | Adopted | Cholesterol, cholesteryl sulphate | HNSC |  |  |
| *RORC* | RAR-related orphan receptor-γ | *RORγ* | NR1F3 | Adopted | Retinoic acid | HNSC |  |  |
| *NR1H3* | Liver X receptor-α | *LXRα* | NR1H3 | Adopted | Liver X receptor- like | Oxysterols | BRCA |  |
| *NR1H2* | Liver X receptor-β | *LXRβ* | NR1H2 | Adopted | Oxysterols | Heterodimer | BRCA, SKCM |  |
| *NR1H4* | Farnesoid X receptor | *FXR* | NR1H4 | Adopted | Bile acids | Heterodimer | LIHC, ESCA |  |
| *VDR* | Vitamin D receptor | *VDR* | NR1I1 | Endocrine | Vitamin D receptor-like | Calcitriol (1',25' dihydroxy vitamin D3) | Heterodimer | HNSC, LIHC, COAD, BLCA, LUAD/LUSC |
| *NR1I2* | Pregnane X receptor | *PXR* | NR1I2 | Adopted | Bile acids |  |  |  |
| *NR1I3* | Constitutive androstane receptor | *CAR* | NR1I3 | Adopted | Androstanol, androstenol |  |  |  |
| *Class II: retinoid X receptor-like* | | | | | | | | |
| *HNF4A* | Hepatocyte nuclear factor-4-α | *HNF4α* | NR2A1 | Adopted | Hepatocyte nuclear factor-4 | Fatty acids | Homodimer | COAD |
| *HNF4G* | Hepatocyte nuclear factor-4-γ | *HNF4γ* | NR2A2 | Adopted |  |  |  |  |
| *RXRA* | Retinoid X receptor-α | *RXRα* | NR2B1 | Adopted | Retinoid X receptor | 9-cis-retinoic acid | Heterodimer | BRCA, COAD, SKCM |
| *RXRB* | Retinoid X receptor-β | *RXRβ* | NR2B2 | Adopted | BRCA, COAD, SKCM |  |  |  |
| *RXRG* | Retinoid X receptor-γ | *RXRγ* | NR2B3 | Adopted | BRCA, COAD, LUAD/LUSC, SKCM |  |  |  |
| *NR2C1* | Testicular receptor 2 | *TR2* | NR2C1 | Orphan | Testicular receptor | All-trans retinoic acid | Homodimer/ heterodimer | PRAD |
| *NR2C2* | Testicular receptor 4 | *TR4* | NR2C2 | Adopted |  |  |  |  |
| *NR2E1* | Homologue of the Drosophila tailless gene | *TLX* | NR2E1 | Orphan | TLX/PNR | Monomer/ homodimer | PRAD |  |
| *NR2E3* | Photoreceptor cell specific nuclear receptor | *PNR* | NR2E3 | Orphan | Benzimidazoles |  |  |  |
| *NR2F1* | Chicken ovalbumin upstream promoter transcription factor I | *COUPTF1* | NR2F1 | Orphan | COUP/EAR | Retinol/ATRA | Homodimer/ heterodimer |  |
| *NR2F2* | Chicken ovalbumin upstream promoter transcription factor II | *COUPTF2* | NR2F2 | Orphan |  |  |  |  |
| *NR2F6* | V-erbA-related | *EAR2* | NR2F6 | Orphan |  |  |  |  |
| *Class III: estrogen receptor-like* | | | | | | | | |
| *ESR1* | Estrogen receptor-α | *ERα* | NR3A1 | Endocrine | Estrogen receptor | Estradiols | Homodimer | HNSC, BRCA, BLCA, OV |
| *ESR2* | Estrogen receptor-β | *ERβ* | NR3A2 | Endocrine | Estradiols, 5α-androstane- 3β, 17β-diol | COAD, OV |  |  |
| *ESRRA* | Estrogen-related receptor-α | *ERRα* | NR3B1 | Adopted | Estrogen related receptor | Monomer/ homodimer | OV |  |
| *ESRRB* | Estrogen-related receptor-β | *ERRβ* | NR3B2 | Adopted |  |  |  |  |
| *ESRRG* | Estrogen-related receptor-γ | *ERRγ* | NR3B3 | Adopted | PRAD, OV |  |  |  |
| *NR3C1* | Glucocorticoid receptor | *GR* | NR3C1 | Endocrine | 3-Ketosteroid receptors | Cortisol (hydrocortisone) | Homodimer | BRCA, PRAD |
| *NR3C2* | Mineralocorticoid receptor | *MR* | NR3C2 | Endocrine | Aldosterone |  |  |  |
| *PGR* | Progesterone receptor | *PR* | NR3C3 | Endocrine | Progesterone | BRCA, OV |  |  |
| *AR* | Androgen receptor | *AR* | NR3C4 | Endocrine | Testosterone, dihydrotesterone | HNSC, BRCA, BLCA, PRAD, LUAD/LUSC |  |  |
| *Class IV: nerve growth factor IB-like* | | | | | | | | |
| *NR4A1* | Nerve Growth factor IB | *NGFIB/NUR77* | NR4A1 | Adopted | NGFIB/NURR1/ NOR1 | Monomer/ homodimer/ |  |  |
| *NR4A2* | Nuclear receptor related 1 | *NURR1* | NR4A2 | Adopted | BLCA, PRAD |  |  |  |
| *NR4A3* | Neuron-derived orphan receptor 1 | *NOR1* | NR4A3 | Adopted |  |  |  |  |
| *Class V: steroidogenic factor-like* | | | | | | | | |
| *NR5A1* | NR5A1 | *SF1* | NR5A1 | Adopted | SF1/LRH1 | Phospholipids | Monomer |  |
| *NR5A2* | NR5A2 | *LRH1* | NR5A2 | Orphan | BRCA, COAD |  |  |  |
| *Class VI: germ cell nuclear factor-like* | | | | | | | | |
| *NR6A1* | NR6A1 | *GCNF* | NR6A1 | Orphan | GCNF | Homodimer |  |  |
| *Class 0: Miscellaneous* | | | | | | | | |
| *NR0B1* | NR0B1 | *DAX1* | NR0B1 | Orphan | DAX/SHP | Heterodimer | PRAD |  |
| *NR0B2* | NR0B2 | *SHP* | NR0B2 | Orphan | CD437 Retinoids | LIHC, KIRC/KIRP |  |  |
